# Fibrillary Glomerulonephritis in a Patient With Vulvar Squamous Cell Carcinoma

**DOI:** 10.7759/cureus.35068

**Published:** 2023-02-16

**Authors:** Ashwin Jagadish, Venkata Vedantam, Neethu Vedantam, Hezborn M Magacha

**Affiliations:** 1 Internal Medicine, East Tennessee State University Quillen College of Medicine, Johnson City, USA; 2 Infectious Diseases, East Tennessee State University Quillen College of Medicine, Johnson City, USA; 3 General Practice, East Tennessee State University College of Public Health, Johnson City, USA

**Keywords:** dnajb9, oncology, nephrology, fibrillary glomerulonephritis, vulvar squamous cell carcinoma, malignancy

## Abstract

Fibrillary glomerulonephritis is a rare condition characterized by glomerular accumulation of non-branching fibrils, leading to hematuria, proteinuria, and hypertension. It is often associated with malignancy but has no known cause. A 66-year-old Caucasian female with a history of vulvar squamous cell carcinoma presented with one month of painless hematuria in the setting of new-onset progressive renal dysfunction and nephrotic range proteinuria. Comprehensive evaluation, including renal biopsy with staining for DnaJ heat shock protein family member B9 (DNAJB9), provided a definitive diagnosis of fibrillary glomerulonephritis. The patient initially received rituximab and prednisone therapy. Unfortunately, her renal function continued to decline over the next month, requiring re-hospitalization with initiation of hemodialysis. To our knowledge, this is the first case describing an association between vulvar squamous cell carcinoma and fibrillary glomerulonephritis.

## Introduction

Fibrillary glomerulonephritis (FGN) is a rare condition found in less than 1% of native renal biopsies and has unclear pathophysiology [1,2]. Individuals with FGN are often older, Caucasian, and female [2,3]. They present with high-grade proteinuria, hematuria, edema, and renal insufficiency (creatinine >1.5 mg/dL, systolic blood pressure >140 mmHg, albumin <3.5 g/dL) [3]. On biopsy, FGN is characterized by capillary wall and/or mesangial deposits of randomly arranged fibrils 10-30 nm in length [4]. Fibrils are thought to be amyloid like and comprise immune complexes; however, they are larger, non-branching, and typically do not stain with Congo red, which contrasts from deposits in amyloid renal disease [2,5]. Diagnosis is confirmed by DnaJ heat shock protein family member B9 (DNAJB9) staining and by viewing fibrils in renal biopsy specimens under an electron microscope [6].

Clinically, FGN is associated with malignancies, dysproteinemias, autoimmune conditions, and hepatitis C [4]. Specific malignancies linked to FGN include multiple myeloma, hepatocellular carcinoma, melanoma, renal cell carcinoma, and cancers of the thyroid, breast, ovary, uterus, biliary tract, prostate, and colon [2,4,7]. To our knowledge, this is the first case report demonstrating an association between vulvar squamous cell carcinoma and fibrillary glomerulonephritis.

## Case presentation

A 66-year-old Caucasian female with hypertension, chronic obstructive pulmonary disease, tobacco dependence, and a history of vulvar squamous cell carcinoma status after radiation therapy was sent to the emergency department from her primary care physician’s office with a progressive worsening kidney injury. The patient was initially diagnosed with vulvar squamous cell carcinoma five years prior for which she underwent radical excision with inguinal lymphadenectomy and CO_2_ laser treatment. Subsequently, she had multiple relapses for which she received wide local excision and adjuvant radiation. The last dose of radiation was two and half years prior to admission. At the time of presentation, the patient had a recently discovered vulvar lesion with biopsy indicating lichen sclerosus et atrophicus. The biopsy results did not indicate malignancy, so no further treatment of the carcinoma was deemed necessary during this admission.

In the emergency department, the patient’s blood pressure was 159/93 mmHg, and physical examination revealed bilateral lower extremity edema. Laboratory studies showed a creatinine level of 2.84 mg/dL, blood urea nitrogen (BUN) 33 mg/dL, and urinalysis significant for large-volume hematuria, proteinuria, and microalbuminuria. The urine protein to creatinine ratio was found to be 3.9 mg/gm consistent with nephrotic range proteinuria. These findings were especially concerning in light of a baseline creatinine level of 1.10 mg/dL five months earlier. The laboratory and urine studies are summarized in Table 1.

**Table 1:** Summary of significant laboratory values ANA, antinuclear antibody; ANCA IFA, antineutrophil cytoplasmic antibody immunofluorescence assay

Laboratory tests	Value	Normal values
Immunologic workup		
ANA IgG	None detected	None detected
Rheumatoid factor (IU/mL)	<10	0-15
Glomerular basement antibody (AU/mL)	0	0-19
Myeloperoxidase antibody (AU/mL)	1	0-19
ANCA IFA titer	<1:20	<1:20
ANCA IFA pattern	None detected	None detected
Qualitative cryoglobulin	Negative at 72 hours	Negative at 72 hours
C3 (mg/dL)	94	87-200
C4 (mg/dL)	37	19-52
Infectious workup		
Hepatitis A antibody, IgM	Non-reactive	Non-reactive
Hepatitis B surface antigen	Non-reactive	Non-reactive
Hepatitis B core antibody, IgM	Non-reactive	Non-reactive
Hepatitis B surface antibody, quantitative (mIU/mL)	0.1	<8.0
Hepatitis C antibody	Non-reactive	Non-reactive
HIV 1/2 antibody or p24 antigen	Non-reactive	Non-reactive
Myeloma workup		
Kappa free light chain (mg/L)	87.06	3.30-19.40
Lambda free light chain (mg/L)	47.17	5.71-26.30
Kappa/lambda free light chain ratio	1.85	0.26-1.65
Serum protein electrophoresis	No monoclonal paraproteins	No monoclonal paraproteins
Urinalysis		
Gross blood	Large	Negative
Protein level (mg/dL)	300	Negative
RBC (/hpf)	≥100	0-4
Microalbumin (mg/dL)	114.6	0.0-1.8
Protein/creatinine ratio	3.9	<0.15
Albumin/creatinine ratio (mg/g)	2292.0	0.0-29.9

**Figure 1:**
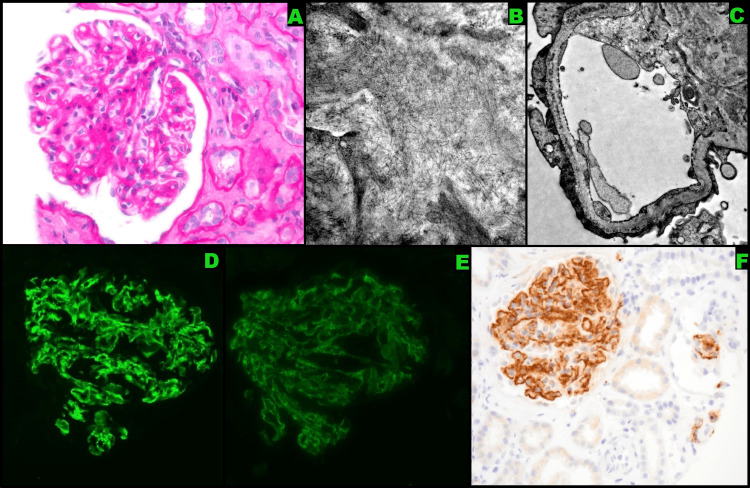
Diagnostic imaging (A) Glomeruli showing mesangial expansion, (B) electron microscopy revealing mesangial fibrils, (C) electron microscopy revealing capillary loop fibrils, (D) glomeruli revealing a positive reaction to IgA, (E) glomeruli revealing a positive reaction to IgG, (F) glomeruli revealing a positive reaction to DnaJ heat shock protein family member B9 (DNAJB9)

The nephrology team was consulted and the patient was started on intravenous empiric pulse dose methylprednisolone 500 mg daily. Patient’s creatinine level continued to worsen despite steroids, so a renal biopsy was performed. The glomeruli demonstrated mesangial expansion and randomly arranged non-branching fibril deposition (Figures 1A-1C). These fibrils were also observed within capillary loops. Immunofluorescence of the glomeruli stained positive for IgA and IgG (Figures 1D-1E). DNAJB9 staining also returned positive (Figure 1F). These findings confirmed the diagnosis of FGN. An extensive workup including hepatitis C, multiple myeloma, and autoimmune disorders was negative.

After ruling out tuberculosis with a negative QuantiFERON Gold test and methicillin-resistant *Staphylococcus aureus* (MRSA) with a negative polymerase chain reaction (PCR) test, she was initiated on rituximab with 1 g delivered during inpatient treatment and 1 g delivered during her outpatient treatment. She also received 20 mg prednisone three times daily for one month. As prophylaxis while on the immunosuppressive regimen, she took trimethoprim-sulfamethoxazole three times per week.

One month after the initial hospitalization, the patient returned with worsening renal function. Her creatinine level was 6.29 mg/dL on admission, so dialysis was initiated. She was discharged the following week, and remains hemodialysis dependent.

## Discussion

This case of fibrillary glomerulonephritis in a patient with vulvar squamous cell carcinoma highlights a rare but rapidly progressive disease. The carcinoma was diagnosed five years prior to the diagnosis of FGN, with multiple relapses along the way. FGN, despite being uncommon, is known to be associated with malignancies and should be considered early within the differential diagnosis in patients with a cancer history. FGN is known to occur in patients who have had malignancy identified up to 15 years prior to the onset of renal disease [7]. Malignancy is present in approximately 9% of patients with FGN [8]. Other diseases are associated with FGN, albeit with a varying frequency. These include include hepatitis C (7%-27%), dysproteinemia (4%-42%), autoimmune diseases (11%), and diabetes mellitus (24%) [8]. Given the incidence of these conditions, we recommend screening for them in patients with FGN. Our patient had negative results for autoimmune disorders, hepatitis C, diabetes mellitus, and dysproteinemia. However, she did have a history of vulvar squamous cell carcinoma, and malignancies are known to be associated with FGN.

One month prior to admission, the patient’s chest CT was unremarkable. During the current admission, the patient had a bladder ultrasound, chest x-ray, and a CT scan of the abdomen and pelvis, which did not show any new malignancy. Her most recent age-appropriate cancer screening including mammogram performed one year ago was normal. A colonoscopy performed after this hospitalization showed only a tubular adenoma. She had a new vulvar lesion that was biopsied due to concern for malignancy; however, no malignancies were detected. A decision was made by the oncologic surgeon to closely monitor the patient with additional biopsies as new lesions arose.

DNAJB9, a cochaperone for heat shock protein 70, is a recently discovered biomarker that is 98% sensitive and 99% specific for identifying FGN [6]. It has now become the gold standard for diagnosing FGN. DNAJB9 is preferentially found in organs with a well-developed endoplasmic reticulum, such as the kidney and liver. There are two proposed mechanisms related to the involvement of DNAJB9 in FGN [6]. The first theory speculates that DNAJB9 becomes deposited in the kidney and forms fibrils. Autoantibodies, such as IgG4, then form against the DNAJB9 molecule. The second theory proposes that DNAJB9 binds to misfolded IgG secondarily. It is possible that individuals with certain predisposing conditions (e.g. malignancy) can develop increased levels of misfolded IgG molecules, thus facilitating DNAJB9 binding [6]. A positive stain is diagnostic of FGN [2,6].

Approximately 50% of individuals with FGN often progress to end-stage renal disease within two years of diagnosis [2,9]. In general, steroid therapy can be provided concomitantly with either rituximab or cyclophosphamide. Renal transplantation can be pursued, but there is a risk of relapse [8]. Rituximab has been viewed as a treatment option for FGN due to findings of polyclonal immunoglobulin deposits in the mesangium and glomerular basement membrane [9]. Rituximab therapy has demonstrated a range of results, from complete to partial response, non-progression, persistent renal dysfunction, progression to end-stage renal disease, or no response at all. Steroid therapy in patients with biopsy-determined FGN may improve renal function if started early [10]. Given the rapidly progressive nature of FGN, a combination of rituximab and prednisone was chosen for this patient due to its potential for a favorable response. It remains possible that the diagnosis of FGN was made too late for this medication regimen to be effective, as noted by the continued rapid decline in renal function and progression to dialysis. Currently, there is no definitive treatment for FGN based on renal function. Additional research is required to identify the ideal treatment for fibrillary glomerulonephritis at different stages of disease progression.

## Conclusions

To our knowledge, this case report is the first to describe fibrillary glomerulonephritis in a patient with a history of vulvar squamous cell carcinoma. The patient had hematuria, proteinuria, renal insufficiency, and edema, all of which are signs of FGN. The condition is rapidly progressive, and renal function can deteriorate rapidly, even resulting in the need for hemodialysis. Rituximab and prednisone are viable treatment options; however, further studies are required to evaluate their efficacy, especially at later stages of disease. This case underscores the importance of early consideration and diagnosis of fibrillary glomerulonephritis in patients with malignancies, particularly in cases of vulvar squamous cell carcinoma.
